# Creating China’s Biosimilar Drugs Regulatory System: A Calculated Approach

**DOI:** 10.3389/fphar.2022.815074

**Published:** 2022-02-02

**Authors:** Jianhong Yang, Xiaoyu Zhao, Jie Li, Kejian Zhang, Zheru Zhang, Shunwen Chang, Daotian Fu, Xinhuan Lyu, Xianglin Zhang, Ying Shao

**Affiliations:** ^1^ Research center of Yeehong Business School, Shenyang Pharmaceutical University, Beijing, China; ^2^ Overland Pharma (Shanghai) Limited Company, Shanghai, China; ^3^ School of Pharmaceuticals, Sun Yat-sen University, Guangzhou, China; ^4^ South China Center for Innovative Pharmaceuticals, Guangdong, China; ^5^ I-Mab, Shanghai, China; ^6^ Skyline Therapeutics, Shanghai, China; ^7^ RemeGen, Ltd., Shandong, China

**Keywords:** biosimilar drug, supervision, law and regulation, safety, efficacy

## Introduction

A biosimilar drug contains active substances similar to the original biologic drug and is similar to the original drug in terms of quality, safety, and effectiveness, with no clinically significant differences (Center for Drug Evaluation of the National Medical Products Administration, 2015). Compared with their original drugs, biosimilar drugs have a price advantage, which is an important approach to improve patient drug accessibility and control medical costs (Jensen et al., 2020; Shan et al., 2020). Given the uniqueness and complexity of biological products, biosimilar drugs are different from chemical generic drugs, and there are significant differences in their review and approval paths. In order to standardize the research and development (R&D) and the review of biosimilar drugs, more than 20 countries worldwide have formulated guidelines related to biosimilar drugs and clear approval paths for marketing them (European Medicines Agency, 2011; European Medicines Agency, 2014a; Shan et al., 2020).

## Necessity to Establish China’s Biosimilar Drug Regulatory System

In recent years, China’s biosimilar drug industry has developed rapidly. By the end of 2019, China had the highest number of biosimilar drugs under research, with 391 biosimilar drugs in the R&D pipeline. As of 31/12/2020, 11 biosimilar drugs had been approved for marketing in China (Table 1) (Yeehong Business School, 2021). However, the laws and regulations for biosimilar drugs in China, particularly a complete registration management system and supporting policies for the supervision of biosimilar drugs, have not yet been clearly formed. Thus, there is an urgent need for a corresponding regulatory system for biosimilar drugs. While developing the laws and regulatory system, efforts should be made to avoid any deviations that happened in the evaluation of chemical generics and should focus on completing the task in hand in one attempt (Huang et al., 2017).

**TABLE 1:** 11 biosimilar drugs that had been approved in China before December 2020.

Drug Name (INN)	Market authorization holder	Time of approval	Indication
Rituximab Injection	Innovent Biologics (Suzhou) Co., Ltd.	10/09/2020	Non-Hodgkin’s lymphoma, NHL
Rituximab Injection*	Shanghai Fuhong Henlius Biopharmaceutical Co., Ltd.	02/22/2019	Non-Hodgkin’s lymphoma, NHL
Adalimumab Injection	Shanghai Fuhong Henlius Biopharmaceutical Co., Ltd.	12/07/2020	Autoimmune diseases such as ankylosing spondylitis, rheumatoid arthritis and psoriasis
Adalimumab Injection	Innovent Biologics (Suzhou) Co., Ltd.	09/03/2020	Autoimmune diseases such as ankylosing spondylitis, rheumatoid arthritis and psoriasis
Adalimumab Injection	Zhejiang Hisun Pharmaceutical Co., Ltd.	12/06/2019	Autoimmune diseases such as ankylosing spondylitis, rheumatoid arthritis and psoriasis
Adalimumab Injection	Bio-Thera Solutions (Guangzhou), Ltd.	11/04/2019	Autoimmune diseases such as ankylosing spondylitis, rheumatoid arthritis and psoriasis
Trastuzumab Injection	Shanghai Fuhong Henlius Biopharmaceutical Co., Ltd.	08/14/2020	Metastatic breast cancer
Trastuzumab Injection	Sunshine Guojian Pharmaceutical (Shanghai)Co., Ltd.	6/19/2020	Metastatic breast cancer
Bevacizumab Injection	Innovent Biologics (Suzhou) Co., Ltd.	6/17/2020	Advanced, metastatic or recurrent non-small cell lung cancer, metastatic colorectal cancer
Bevacizumab Injection	Qilu Pharmaceutical Co., Ltd.	12/06/2019	Advanced, metastatic or recurrent non-small cell lung cancer, metastatic colorectal cancer
Insulin aspart	Gan & Lee Pharmaceuticals	05/12/2020	Diabetes

## Thoughts and Recommendations on the Creation of a Biosimilar Drug Regulatory System in China

Identifying reference drugs for biosimilar drugs, managing labels, nomenclature, defining interchangeable biological products, and creating a catalog including “a set of marketed biological products” are the management elements that need to be improved urgently in the current stage of China’s biosimilar marketing authorization. We have put forward some suggestions to improve each of the aforementioned elements.

### Identifying Reference Drugs

The reference drug used in the R&D of biosimilar drugs should be an original biological product approved by the Chinese regulatory authority based on clinical safety and efficacy data. There are two recommendations for obtaining reference drugs: reference drugs purchased from the Chinese market and those purchased from the International Council for Harmonisation of Technical Requirements for Pharmaceuticals for Human Use (ICH) national markets approved by China (Wang, 2020).

### Managing Labels

It is difficult to ensure that the macromolecular structural characteristics of biosimilars are completely consistent with those of the originally developed drugs. Therefore, it is necessary to indicate the differences between biosimilar drugs and their originally developed drugs on the label. It is suggested that different instructions regarding the label management requirements should be determined based on the developmental stages of regulation (European Medicines Agency, 2014b; US Food and Drug Administration, 2018).

The label should state that the product is a biosimilar of the reference drug and list the comparative study data of the biosimilar based on the marketing authorization that made the decision. Furthermore, it should list the safety and efficacy data related to the approved reference drug (only including the data corresponding to the approved indications of biosimilar drugs) to provide sufficient information for clinicians to make prescription decisions.

With adjustment in laws and regulations of biosimilar drugs and with maturity of the industry, the contents of the label should be gradually simplified, whereby the label would simply declare that the product is a biosimilar of a reference drug. The contents of the label would be exactly the same as those for the reference drug, without the clinical research comparison data for biological similarity.

### Extrapolating Indications

China has issued the extrapolation of biosimilar drugs, and it clarifies the prerequisites for the extrapolation of indications of biosimilar drugs (Center for Drug Evaluation of the National Medical Products Administration, 2021). On this basis, it is suggested that the extrapolated indications should be those approved for marketing in China in order to reduce the unknown risks posed by ethnic differences. At the same time, declaration of all indications of the original biological products should be encouraged when marketed in China, in order to provide a reference basis for the extrapolation of the indications of their biosimilar drugs.

### Nomenclature

Based on the nature of biosimilar drugs, it is determined that immunogenicity is a unique safety issue, and it is difficult for biosimilar drugs to be completely consistent with the reference drugs (National Pharmacopoeia Commission of China, 2018; Guo, 2020). In addition, the indications of biosimilar drugs may be different from those of their reference drugs. Distinct names will help attract the attention of clinicians. For accurate pharmacovigilance management throughout the life cycle of biosimilar drugs, “distinguishability” should be the primary principle in naming biosimilar drugs. It may be more desirable to define biosimilar with a trade name which can encourage brand building of enterprises and promote the quality improvement of biosimilar drugs. However, the name method is difficult to be implemented in China currently because of violating the current prescription management measures (Ministry of Health of the People’s Republic of China, 2006). It is worth expecting to establish a more mature policy environment for biosimilars.

### Interchangeability of Biosimilar Drugs

Considering the safety risks associated with the immunogenicity of biosimilar drugs, there are some potential risks in the use of completely interchangeable substitutes for the reference drugs (Liu and Zhang, 2020). As China’s biosimilar drug industry is still in its initial stage, it is suggested that the establishment of “interchangeable biosimilar drugs” (Food and Drug Administration, 2009) should not be considered in the market authorization review stage. It can be decided by the physicians whether to replace a drug with another drug with the same therapeutic purpose (also called switching) (European Biopharmaceutical Enterprises (EBE) and European Federation of Pharmaceutical Industries Associations (EFPIA), The European Federation of Pharmaceutical Industries and Associations (IFPMA), 2017, (EBE-EFPIA and IFPMA, 2017). Physicians prescribe appropriate biosimilar drugs based on their understanding of these drugs and the development of the patient’s condition.

### Creating a Catalog of Marketed Biosimilar Drugs

China should establish a catalog of marketed biosimilar drugs to further encourage the development of the biosimilar drug industry and provide necessary information for the general public and health care professionals. The catalog can be consistent with that of marketed chemical drugs in China, including approval information, designated reference drugs, and data protection information of the reference drug (Yang et al., 2019).

## Discussion

There are something that need further discussion. On one hand, We know the price differences between biosimilars and the originators may be limited difference in some countries, we also need to consider the factors which have impact on the price in terms of competition and local production (Godman et al., 2021). On the other hand, we would suggest a series of demand-side efforts to improve the confidence of both physicians and consumers on biosimilars (Kim et al., 2020). Furthermore, the real world studies could provide clinical evidences (Colloca et al., 2019) to support the switch from originators to biosimilars (Jørgensen et al., 2017).

With the acceleration of global economic integration and China’s entry into the ICH community, the scientific principles, methods, and requirements followed by China’s regulatory system for biosimilar drugs are being gradually aligned to international standards. China has issued technical guidelines for R&D, clinical trials, and the evaluation of biosimilar drugs. However, there are still many challenges in establishing a systematic and complete biosimilar drug regulatory system.

First, China’s unique historical issues have brought challenges to the regulation of biosimilar drugs. Owing to the lag in China’s biosimilar drug regulatory system and the incomplete technical guidance system, there are some products that were approved *via* the new drug review pathway, which requires non-comparable research, and these non-comparable products are on the market. These deficiencies will add difficulties to the marketing authorization and post-marketing supervision of biosimilar drugs. Therefore, it is necessary to balance the development of biosimilar drugs and historical problems.

Second, Biosimilar drugs also face challenges in pricing and reimbursement. It is necessary to issue relevant incentive policies to ensure the fairness of market access. It is necessary to set reasonable prices on the basis of comprehensive enterprise R&D investment and guarantee product quality in order to promote the development of the biosimilar drug industry. Whether in China or European or American countries, greater price reduction may be the problem that biosimilar drugs need to address directly (Zhao, 2019).

Third, it is a dilemma to find a proper naming method of biosimilars because biosimilars are not allowed to be prescribed with brand name according to current prescription management measures in China. It is suggested to set up a more mature policy environment to support an appropriate way of biosimilars’ nomenclature.
